# High Densities of Tumor-Associated Plasma Cells Predict Improved Prognosis in Triple Negative Breast Cancer

**DOI:** 10.3389/fimmu.2018.01209

**Published:** 2018-05-30

**Authors:** Joe Yeong, Jeffrey Chun Tatt Lim, Bernett Lee, Huihua Li, Noel Chia, Clara Chong Hui Ong, Weng Kit Lye, Thomas Choudary Putti, Rebecca Dent, Elaine Lim, Aye Aye Thike, Puay Hoon Tan, Jabed Iqbal

**Affiliations:** ^1^Division of Pathology, Singapore General Hospital, Singapore, Singapore; ^2^Singapore Immunology Network (SIgN), Agency of Science, Technology and Research (A*STAR), Singapore, Singapore; ^3^Division of Medicine, Singapore General Hospital, Singapore, Singapore; ^4^Faculty of Medicine, University of New South Wales, Sydney, NSW, Australia; ^5^Centre for Quantitative Medicine, Duke-NUS Medical School, Singapore, Singapore; ^6^Department of Pathology, Yong Loo Lin School of Medicine, National University of Singapore, Singapore, Singapore; ^7^National Cancer Center, Singapore, Singapore

**Keywords:** triple negative breast cancer, plasma cells, B cells, immunohistochemistry, tumor immunology

## Abstract

Breast cancer is the most common malignancy affecting women, but the heterogeneity of the condition is a significant obstacle to effective treatment. Triple negative breast cancers (TNBCs) do not express HER2 or the receptors for estrogen or progesterone, and so often have a poor prognosis. Tumor-infiltrating T cells have been well-characterized in TNBC, and increased numbers are associated with better outcomes; however, the potential roles of B cells and plasma cells have been large. Here, we conducted a retrospective correlative study on the expression of B cell/plasma cell-related genes, and the abundance and localization of B cells and plasma cells within TNBCs, and clinical outcome. We analyzed 269 TNBC samples and used immunohistochemistry to quantify tumor-infiltrating B cells and plasma cells, coupled with NanoString measurement of expression of immunoglobulin metagenes. Multivariate analysis revealed that patients bearing TNBCs with above-median densities of CD38^+^ plasma cells had significantly better disease-free survival (DFS) (HR = 0.44; 95% CI 0.26–0.77; *p* = 0.004) but not overall survival (OS), after adjusting for the effects of known prognostic factors. In contrast, TNBCs with higher immunoglobulin gene expression exhibited improved prognosis (OS *p* = 0.029 and DFS *p* = 0.005). The presence of B cells and plasma cells was positively correlated (*p* < 0.0001, *R* = 0.558), while immunoglobulin gene IGKC, IGHM, and IGHG1 mRNA expression correlated specifically with the density of CD38^+^ plasma cells (IGKC *p* < 0.0001, *R* = 0.647; IGHM *p* < 0.0001, *R* = 0.580; IGHG1 *p* < 0.0001, *R* = 0.655). Interestingly, after adjusting the multivariate analysis for the effect of intratumoral CD38^+^ plasma cell density, the expression levels of all three genes lost significant prognostic value, suggesting a biologically important role of plasma cells. Last but not least, the addition of intratumoral CD38^+^ plasma cell density to clinicopathological features significantly increased the prognostic value for both DFS (ΔLRχ^2^ = 17.28, *p* = 1.71E−08) and OS (ΔLRχ^2^ = 10.03, *p* = 6.32E−08), compared to clinicopathological features alone. The best combination was achieved by integrating intratumoral CD38^+^ plasma cell density and IGHG1 which conferred the best added prognostic value for DFS (ΔLRχ^2^ = 27.38, *p* = 5.22E−10) and OS (ΔLRχ^2^ = 21.29, *p* = 1.03E−08). Our results demonstrate that the role of plasma cells in TNBC warrants further study to elucidate the relationship between their infiltration of tumors and disease recurrence.

## Introduction

Breast cancer is the most common malignancy in women, affecting approximately 12% of females during their lifetimes (1). The disease exists as numerous heterogeneous subtypes, with tumors classified based on histological or molecular characteristics, which result in distinct biological characteristics and clinical prognoses. Triple negative breast cancers (TNBCs), defined by the absence of hormone receptor (estrogen receptor, progesterone receptor) and cerbB2 (HER2) expression, constitute about 15–20% of all breast cancers (2–4). They pose significant management challenges due to the lack of effective treatment options and often exhibit aggressive clinical behavior (5–9). There is an urgent need to better understand the processes driving progression in these tumors, and to define biomarkers that can transcend their heterogeneity and enable optimal and individualized treatment strategies.

Compared to other breast cancer subtypes, TNBCs exhibit more abundant lymphoid cell infiltrates; various components of which have been variably correlated with better or worse prognosis (10–15). More recent studies have revealed the importance of considering the broader picture of immune cell infiltrates: our group showed that the density of Foxp3^+^ regulatory T cells is closely correlated with the abundance of CD8^+^ cytotoxic T cells, and that a high frequency of both cell types predicts better disease-free survival (DFS) and overall survival (OS) in TNBC (16); while Bottai et al. demonstrated that the density of total tumor-infiltrating lymphocytes (TILs) had superior prognostic value compared to the density of CD4^+^, CD8^+^, or FOXP3^+^ lymphocytes, or the expression level of PD-1 or LAG-3 on lymphocytes (17). Thus future progress will require us to understand the full suite of immune cells present in the tumor, their relative abundance, and their functional profile, in order to paint a complete picture of tumor-immune interactions and their effects on disease outcome.

Although both humoral and cellular arms of the immune system are involved in the development and progression of tumors (18–22), relatively few studies have focused on the role of tumor-infiltrating B cells in tumorigenesis. Furthermore, the success of monoclonal antibody based immunotherapy indicates the potential for harnessing the humoral immune response in breast cancer treatment (21, 23–28). Some studies found that B cells were present and activated in approximately one quarter of breast tumors, and represented up to 40% of the TIL population in some (29–32); in one study, B cells were detected early during tumor development (33, 34), and expression of the B cell marker CD20 was significantly elevated in TNBC compared to other breast cancer subtypes (35).

Following antigen exposure and T cell licensing, B cells differentiate into potent antibody-secreting plasma cells, which no longer express CD20 (36), but can be distinguished by expression of CD38 (20, 21, 37–39). Tumor-infiltrating plasma cells were first reported in the 1980s (40), but have not been well-studied in TNBC. Some studies suggest that higher frequencies of CD138^+^ B cells, which might be plasma cells, are linked with poorer recurrence-free survival in breast cancers (41); and breast cancers in which 50% or more of stromal TILs are plasma cells were found to have significantly worse disease-free and OS (42). However, molecular studies draw a contradictory conclusion: high expression of groups of B cell/plasma cell genes (collectively termed “metagenes”) has been associated with significantly better prognosis and response to chemotherapy in breast cancer (43–46). In order to make sense of these disparate conclusions, we need to understand the relationship between B cell and plasma cell infiltration in breast cancer, and to examine expression of B cell/plasma cell-related genes in the context of immunohistochemical data.

In this study, we assessed the frequency and localization of B cells and plasma cells within samples from 269 TNBC tumors; we then measured expression of B cell/plasma cell-related genes within matched samples, and asked how these factors were associated with each other and with clinical outcome.

## Materials and Methods

### Patients and Tumors

A total of 269 archival formalin-fixed paraffin-embedded (FFPE) TNBC specimens from patients diagnosed between 2003 and 2010 at the Department of Anatomical Pathology, Division of Pathology, Singapore General Hospital were analyzed. All samples were obtained before patients underwent chemo- or radio-therapy. Clinicopathological parameters, including patient age, tumor size, histologic growth pattern, grade, and subtype, associated with ductal carcinoma *in situ*, lymphovascular invasion, and axillary lymph node status were reviewed (Table S1 in Supplementary Material). The age of patients ranged between 28 and 89 years (median 55 years); length of follow-up ranged from 1 to 213 months (mean 101, median 97), with recurrence and death occurring in 29 and 24% of these women, respectively. Tumors were typed, staged, and graded according to World Health Organization, American Society of Clinical Oncology—College of American Pathologists (ASCO-CAP) guidelines (47). The Centralized Institutional Review Board of SingHealth provided ethical approval for the use of patient materials in this study (CIRB Ref: 2013/664/F and 2015/2199).

### Tissue Microarray (TMA) Construction

Tumor regions for TMA construction were selected based on pathological assessment of >50% of the sample being tumor area. For each sample, two or three representative tumor cores of 1 mm diameter were transferred from donor FFPE tissue blocks to recipient TMA blocks using a MTA-1 Manual Tissue Arrayer (Beecher Instruments, Sun Prairie, WI, USA). TMAs were constructed as previously described (7).

### Immunohistochemistry Analysis of TMAs

Tissue microarray sections of 4 µm thickness were incubated with antibodies specific for CD3, CD8, CD20, CD38, and Foxp3, as well as ER, PR, and HER2 (Table S2 in Supplementary Material). We also labeled some sections with antibodies specific for epidermal growth factor receptor (EGFR), cytokeratins (CK) 14, and high molecular weight (clone 34βE12) to identify TNBCs that possess the basal-like phenotype, according to previously published protocols (7, 48). Appropriate positive and negative controls were included. Scoring of antibody-labeled sections was carried out for nuclear ER and PR, membrane HER2 and EGFR, and cytoplasmic CK14 and 34βE12 positivity. To generate the score, images of labeled slides were captured using a ScanScope XT device (Aperio Technologies, Vista, CA, USA) or an IntelliSite Ultra-Fast Scanner (Philips, Eindhoven, Netherlands) before viewing by two pathologists blinded to the clinicopathological and survival information. ASCO-CAP guidelines were used to define positivity cutoffs for the tumors: for ER, PR, CK14, EGFR, and 34βE12, a positive result was defined by the presence of at least 1% of tumor cells displaying any intensity of unequivocal staining, and for HER2, tumor positivity was defined by more than 10% of tumor cells exhibiting 3^+^ membrane staining (49). Equivocal HER2 cases were tested and confirmed by fluorescence *in situ* hybridization based on the ASCO/CAP guidelines (50, 51).

Tumor-infiltrating lymphocytes expressing CD20 (B cells) or CD38 (plasma cells) were identified within the stromal and intratumoral regions separately. Plasma cells were presented as the percentage of the intratumoral or stromal areas occupied by the respective cell population, based on published methods (52, 53). Tumors were then divided into “high” and “low” with respect to a particular cell population, when the percentage of the intratumoral or stromal areas occupied by cells labeled for either CD38 (plasma cells) or CD20 (B cells) was above or on/below the median, respectively. Furthermore, cutoff median percentages used were also compatible to the accepted clinical pathological practices: 5% for intratumoral CD38^+^ plasma cells and CD20^+^ B cells, and 1% for stromal CD38^+^ plasma cells and CD20^+^ B cells.

### RNA Extraction, NanoString Measurement of Gene Expression, and Analysis

RNA was extracted from unlabeled FFPE sections of 10 µm thickness using the RNeasy FFPE kit (Qiagen, Hilden, Germany) on a QIAcube automated sample preparation system (Qiagen, Hilden, Germany) and was quantified by an Agilent 2100 Bioanalyzer system (Agilent, Santa Clara, CA, USA). A total of 100 ng of functional RNA (>300 nucleotides) was assayed on the nCounter MAX Analysis System (NanoString Technologies, Seattle, WA, USA). The NanoString counts were normalized using the positive control probes as well as the housekeeping genes, as previously reported (16). The count data were then logarithmically transformed prior to further analysis. *p* Values <0.05 were deemed to be statistically significant.

### Gene Heat Map, Validation, Follow-Up, and Statistical Analysis

Follow-up data were obtained from medical records. DFS and OS were defined as the time from diagnosis to recurrence or death/date of last follow-up, respectively. Statistical analysis was performed using SPSS for Windows, Version 23. The relationship between clinicopathological parameters and the frequency of CD38^+^ plasma cells and CD20^+^ B cells was tested using χ^2^ and Fisher’s exact tests. Survival outcomes were estimated with the Kaplan–Meier analysis and compared between groups with log-rank statistics. Multivariate Cox regression was carried out to evaluate the effect of various tissue compartmentalization of CD38 and CD20 status, as well as NanoString counts of *IGKC, IGHM*, and *IGHG1*, with survival after adjusting for clinicopathological parameters, including patient age, tumor size, tumor grade, and lymph node status. Models were compared using the reduction in the log-likelihood of the models (ΔLRχ^2^) using a likelihood ratio test. A *p* value <0.05 is defined as statistical significant.

## Results

### High Intratumoral Plasma Cell Density Is Associated With Longer Time to Relapse in TNBC

**Figure 1:**
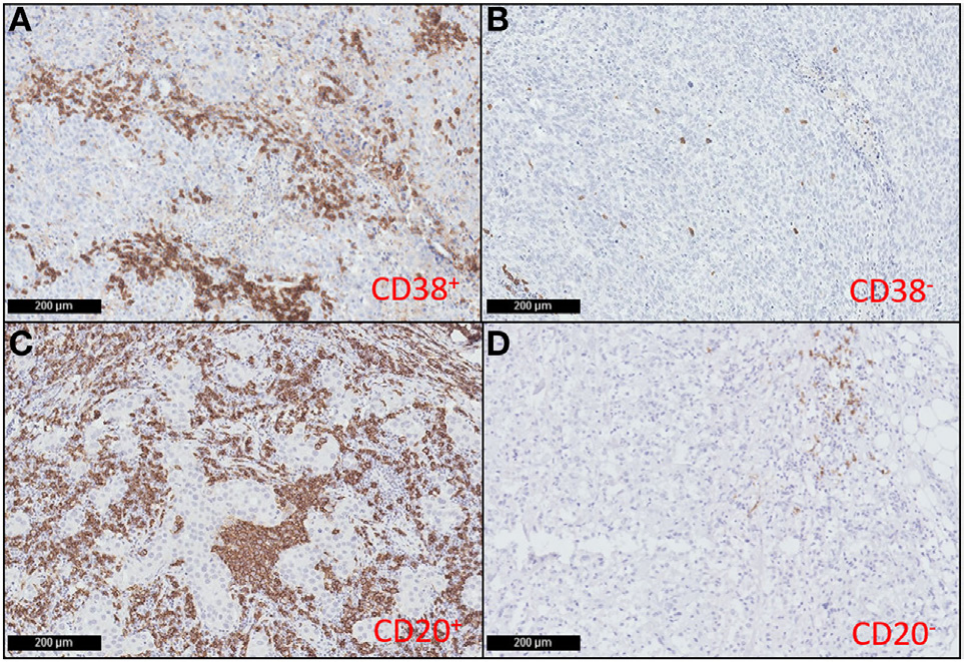
CD38^+^ plasma cells and CD20^+^ B cells infiltrate triple negative breast cancers (TNBC). Representative immunohistochemical staining showing high **(A)** and low **(B)** CD38^+^ plasma cell density; and high **(C)** and low **(D)** CD20^+^ B cell density in TNBC sections (magnification: 100×).

Previous studies have relied upon CD138 as a plasma cell marker, however, as this molecule is also expressed on some tumor cells, we used CD38 to discriminate plasma cells within tumors (54–57). Our previous study showed that the prognostic value of T cells in breast cancer varied depending on their localization within the tumor (16). In this study, we labeled TNBC sections for CD20 or CD38 and quantified the area of positive labeling within the intratumoral and stromal areas separately. Samples were then grouped according to whether their intratumoral or stromal B cell or plasma cell densities were high (above median), or low (on/below median). Representative images of high and low CD38^+^ plasma cell and CD20^+^ B cell TNBC sections are shown in Figure 1. Univariate analyses did not reveal any association between the high/low density of B cells or plasma cells in either the intratumoral or stromal regions with clinicopathological features of the TNBC sample cohort (Table S1 in Supplementary Material), and in agreement with our previous study (16). However, there was clear evidence of a significant positive correlation between the densities of intratumoral CD20^+^ B cells and intratumoral CD38^+^ plasma cells (*p* < 0.0001, *R* = 0.558) (Table S3 in Supplementary Material).

**Figure 2:**
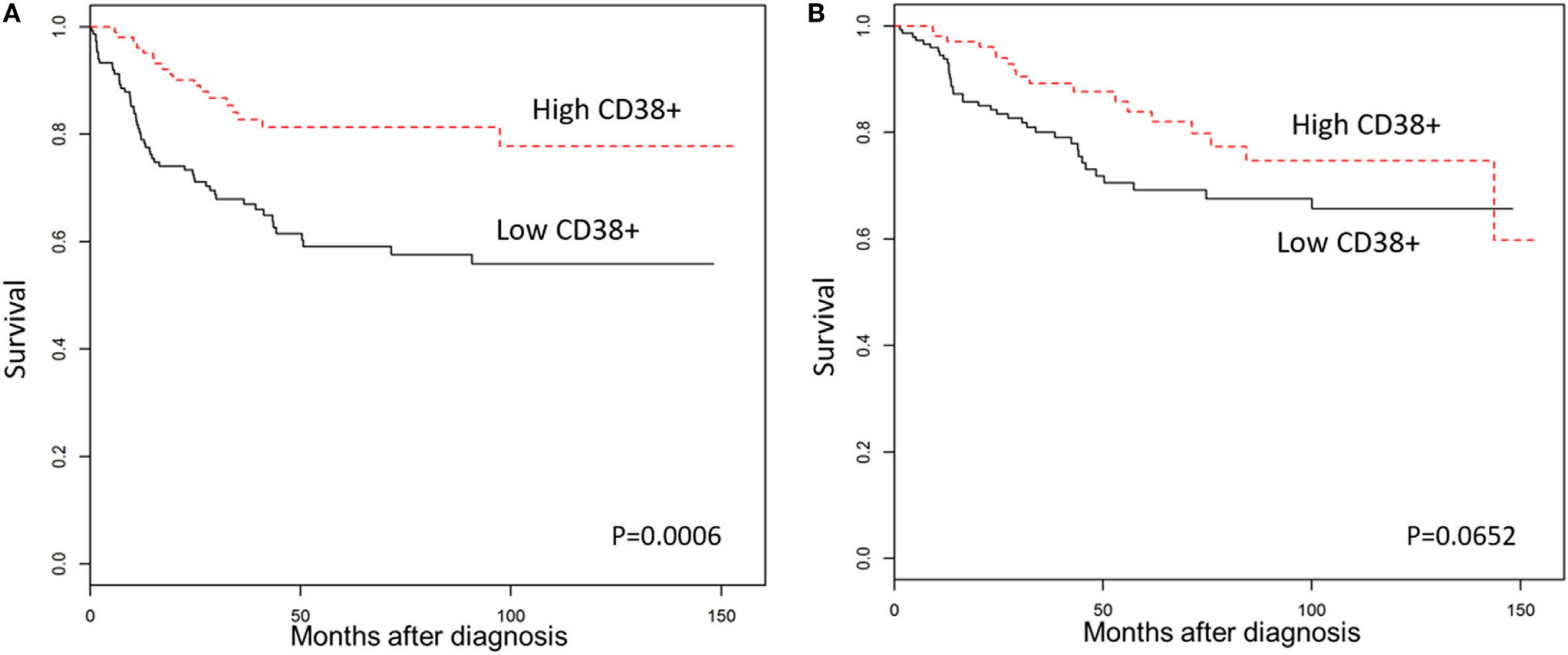
High CD38^+^ plasma cell density is associated with better survival in triple negative breast cancers (TNBC). Kaplan–Meier analysis of survival outcomes in women with high vs. low densities of intratumoral CD38^+^ plasma cells. **(A)** Disease-free survival; **(B)** overall survival.

We then explored the association between plasma cell density of the tumors and the clinical outcomes in TNBC patients. As shown in Figure 2, Kaplan–Meier survival analysis revealed significantly better DFS in TNBC patients within the “high intratumoral CD38^+^ plasma cell” group compared to the “low CD38^+^ intratumoral plasma cell” group (*p* = 0.0006); while OS was not significantly different between groups (*p* = 0.0652), and the density of stromal plasma cells did not affect survival outcomes (Table S4 in Supplementary Material). Multivariate analysis further supported the fact that high density of intratumoral CD38^+^ plasma cells in TNBC was associated with a significantly better DFS (HR = 0.44; 95% CI 0.26–0.77; *p* = 0.004), which was also evident at every 1 percent increment level of CD38^+^ plasma cell denstity (Table 1). In other words, every incremental 1 percent was associated with better DFS (HR = 0.95; 95% CI 0.93–0.98; *p* = 0.002).

**Table 1:** Multivariate analysis of intratumoral CD38^+^ plasma cell and CD20^+^ B cell density with survival outcomes in triple negative breast cancer (TNBC) patients.

	Hazard ratio	95% confidence interval	*p*Value
**Disease-free survival (DFS)**			
Intratumoral CD38^+^ plasma cell TNBCs High vs. low	0.44	(0.26, 0.77)	0.004*
Intratumoral CD38^+^ plasma cell TNBCs (every 1 percent)	0.95	(0.93, 0.98)	0.002*
Intratumoral CD20^+^ B cell TNBCs High vs. low	0.50	(0.25, 0.99)	0.046*
Intratumoral CD20^+^ B cell TNBCs (every 1 percent)	0.98	(0.95, 1.01)	0.228
**Overall survival (OS)**			
Intratumoral CD38^+^ plasma cell TNBCs High vs. low	0.66	(0.36, 1.2)	0.171
Intratumoral CD38^+^ plasma cell TNBCs (every 1 percent)	0.98	(0.96, 1.01)	0.187
Intratumoral CD20^+^ B cell TNBCs High vs. low	0.42	(0.19, 0.97)	0.042*
Intratumoral CD20^+^ B cell TNBCs (every 1 percent)	1.00	(0.96, 1.03)	0.792

*Analysis was adjusted for tumor size, grade, age, and lymph node status*.

*Statistically significant.

Multivariate analysis similarly showed that high intratumoral CD20^+^ B cell density was associated with better OS (HR = 0.42; 95% CI 0.19–0.97; *p* = 0.042) and DFS (HR = 0.50; 95% CI 0.25–0.99; *p* = 0.046), but these effects were not detectable at 1 percent incremental levels (Table 1). Patients with TNBC bearing high densities of CD20^+^ B cells in stromal regions also showed significantly better OS and DFS (Table S4 in Supplementary Material). However, incremental stromal CD20^+^ B cells achieved significance for DFS but not OS.

We then asked about the interaction of intratumoral B cell/plasma cell densities in TNBC by comparing survival outcomes between four combinatorial phenotype groups (Table 2): “High intratumoral CD20^+^ B cell and high CD38^+^ plasma cell TNBCs” (*n* = 33), “High intratumoral CD20^+^ B cell and low CD38^+^ plasma cell TNBCs” (*n* = 17), “Low intratumoral CD20^+^ B cell and high CD38^+^ plasma cell TNBCs” (*n* = 10), and “Low intratumoral CD20^+^ B cell and low CD38^+^ plasma cell TNBCs” (*n* = 33). Multivariate analysis showed that patients having high intratumoral B cell and plasma cell densities had significantly better OS (HR = 0.26; 95% CI 0.09–0.75; *p* = 0.013) and DFS (HR = 0.24; 95% CI 0.09–0.64; *p* = 0.004) compared to patients with low densities of intratumoral B cells and plasma cells (Table 2). When considering the same combinatorial phenotypes for the stromal compartment, only “High stromal CD20^+^ B cells and low CD38^+^ plasma cells TNBCs” were significantly associated with both OS and DFS (Table S5 in Supplementary Material).

**Table 2:** Multivariate analysis of combinatorial intratumoral B cell/plasma cell density phenotypes with survival outcomes in triple negative breast cancers (TNBC).

Reference to: low intratumoral CD20+ B cell and low intratumoral CD38+ plasma cell TNBCs	*N*number 63	Hazard ratio	95% confidence interval	*p*Value
**Disease-free survival (DFS)**				
High intratumoral CD20^+^ B cell and low CD38^+^ plasma cell TNBCs	17	0.71	(0.29, 1.76)	0.460
Low intratumoral CD20^+^ B cell and high CD38^+^ plasma cell TNBCs	10	0.29	(0.07, 1.23)	0.093
High intratumoral CD20^+^ B cell and high CD38^+^ plasma cell TNBCs	33	0.24	(0.09, 0.64)	0.004*
**Overall survival (OS)**				
High intratumoral CD20^+^ B cell and low CD38^+^ plasma cell TNBCs	17	0.34	(0.08, 1.48)	0.150
Low intratumoral CD20^+^ B cell and high CD38^+^ plasma cell TNBCs	10	0.18	(0.02, 1.36)	0.096
High intratumoral CD20^+^ B cell and high CD38^+^ plasma cell TNBCs	33	0.26	(0.09, 0.75)	0.013*

*Statistically significant.

### Intratumoral CD38^+^ Plasma Cell Density Is an Independent Prognostic Marker in TNBC

We previously reported the prognostic influence of intratumoral CD20^+^ B cell and CD3^+^ T cell density in TNBC (16). Therefore, we performed further multivariate analyses on TNBC with high or low intratumoral CD38^+^ plasma cells, adjusted for the effects of B and T cell density. We found that the density of intratumoral CD38^+^ plasma cells was an independent prognostic marker for both DFS (HR = 0.28; 95% CI 0.11–0.71; *p* = 0.007) and OS (HR = 0.28; 95% CI 0.10–0.82; *p* = 0.020) (Table 3). Furthermore, adjusting for the effects of CD20^+^ B cell density similarly identified intratumoral CD38^+^ plasma cell density as an independent prognostic marker (Table S6 in Supplementary Material). One percent incremental increases in CD38^+^ plasma cell density showed significant prognostic value in DFS, but not OS, even after adjustment for the density of CD3^+^ T cells and CD20^+^ B cells (Table 3), or for CD20^+^ B cells alone (Table S6 in Supplementary Material). These findings were confirmed using *CD38* gene expression data from a publicly available database [METABRIC, EGAS00001001753 from the European Genome–phenome Archive (58)], which revealed a significant association between increasing *CD38* expression and both DFS (HR = 0.82; 95% CI 0.68–0.97, *p* = 0.0229) and OS (HR = 0.83; 95% CI 0.72–0.97, *p* = 0.0191) in 320 cases of TNBC (Table 4). Besides the METABRIC cohort (58) we also analyzed gene expression and survival of TNBCs from The Cancer Genome Atlas (59) which was obtained from cBioPortal (60, 61) for validation purposes, after filtering for TNBC samples. However, CD38 gene expression in this TNBC cohort (*n* = 89) is not a prognostic marker (*p* = 0.235) which might be due to the small sample size.

**Table 3:** Multivariate analysis showed that high intratumoral CD38^+^ plasma cell populations are significantly associated with longer disease-free survival (DFS), compared to low intratumoral CD38^+^ plasma cells populations in triple negative breast cancers (TNBCs), with the median cutoff and with every 1 percent increase.

	Hazard ratio	95% confidence interval	*p*Value
**DFS**			
Intratumoral CD38^+^ plasma cells TNBCs High vs. low	0.28	(0.11, 0.71)	0.007*
Intratumoral CD38^+^ plasma cells TNBCs (every 1 percent)	0.95	(0.91, 1.00)	0.031*
**Overall survival (OS)**			
Intratumoral CD38^+^ plasma cells TNBCs High vs. low	0.28	(0.10, 0.82)	0.020*
Intratumoral CD38^+^ plasma cells TNBCs (every 1 percent)	0.96	(0.91, 1.01)	0.126

*Intratumoral CD38^+^ plasma cells are associated with better OS with the median cutoff (multivariate analysis adjusted for tumor size, grade, age, and lymph node status, intratumoral CD20^+^ B cells, and CD3^+^ T cells)*.

*Statistically significant.

**Table 4:** Analysis of *CD38* expression level and survival outcomes in triple negative breast cancers (TNBC) using data from the European Genome–phenome Archive.

	Hazard ratio	95% confidence interval	*p*Value
**Disease-free survival (DFS)**			
*CD38* (every 1 unit)	0.82	(0.68, 0.97)	0.0229*
**Overall survival (OS)**			
*CD38* (every 1 unit)	0.83	(0.72, 0.97)	0.0191*

*Statistically significant.

### Higher Expression of IgG Genes Is Associated With Improved Survival Outcomes in TNBC

**Figure 3:**
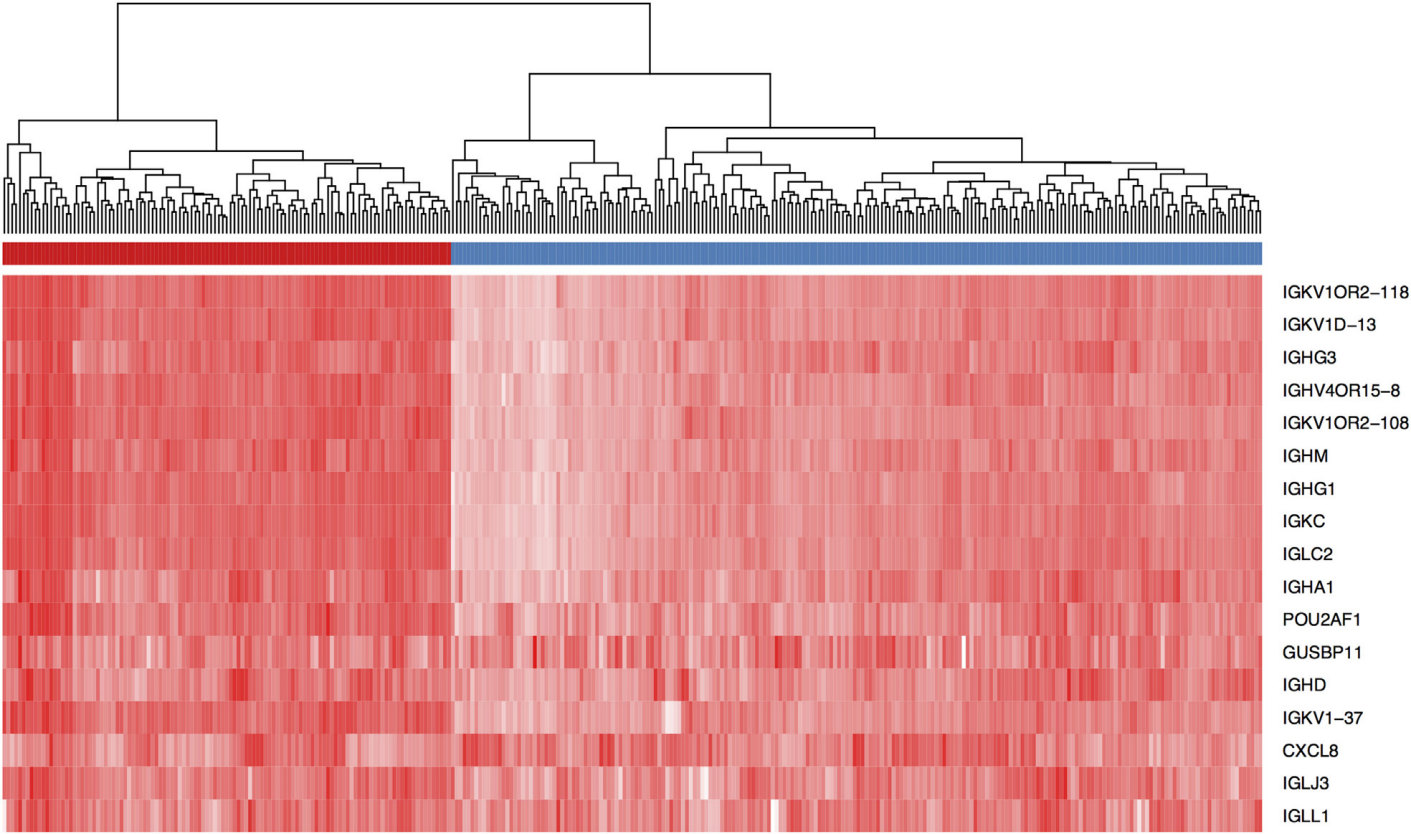
Expression level of a panel of 16 IgG genes and *CXCL8* defines two groups of triple negative breast cancer (TNBC) patients. Unsupervised hierarchical clustering using Euclidean distance revealed the existence of two TNBC patient clusters (red and blue) based on expression intensity of the 17 genes listed. The heat map is colored by the log10 normalized counts with the highest expression in red and the lowest expression in white.

**Figure 4:**
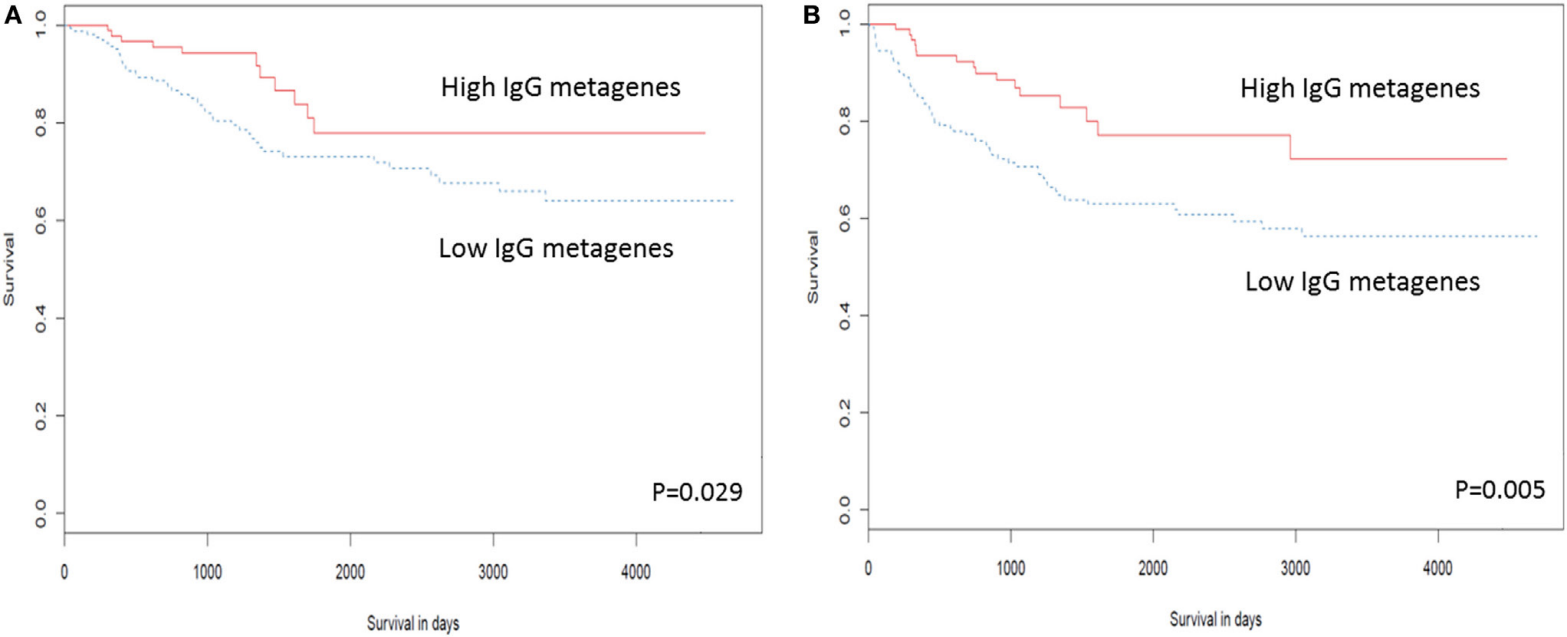
High expression of the IgG metagene is associated with better clinical outcome in triple negative breast cancer (TNBC). Kaplan–Meier analysis of survival outcomes in TNBC in women with high vs. low expression of the IgG metagene, comprising 16 IgG genes and *CXCL8*. **(A)** Disease-free survival; **(B)** overall survival.

Several studies have examined the link between expression of a panel of B cell-related genes, termed metagenes, and breast cancer prognosis (43–46). We selected 16 IgG genes from a previously published metagene panel (62), for which the expression was likely to reflect functions of B cells and plasma cells. Expression levels of 9 of the 16 IgG genes were positively correlated with better OS, while expression of 11 of the 16 was correlated with DFS (Table S7 in Supplementary Material). The combination of these 16 IgG genes with *CXCL8* has been suggested as a prognostic marker in breast cancer in general (44, 63); we, therefore, examined expression of this metagene in our TNBC cohort. Unsupervised hierarchical analysis revealed the existence of two clusters of TNBC (Figure 3): cluster 1 (red) contained TNBC with higher metagene expression and exhibited significantly better OS (*p* = 0.029) and DFS (*p* = 0.005) than did the low metagene-expressing cluster 2 (blue) (Figure 4). Of the IgG genes within the metagene panel, three genes: *IGKC, IGHM*, and *IGHG1*, have been reported to pre-dominate in both lymph node-negative breast cancer, and TNBC (43, 64–66). We found that expression levels of these three genes significantly and positively correlated with the abundance of CD38^+^ plasma cells in our TNBC samples (*IGKC p* < 0.0001, *R* = 0.647; *IGHM p* < 0.0001, *R* = 0.580; *IGHG1 p* < 0.0001, *R* = 0.655, Table S3 in Supplementary Material); and were associated with incrementally increasing DFS in multivariate analysis adjusted for tumor size, grade, age, and lymph node status (Table 5). Increasing *IGHG1* expression was also associated with better OS (Table 5). Interestingly, after adjusting the multivariate analysis for the effect of intratumoral CD38^+^ plasma cell density, the expression levels of all three genes lost significant prognostic value (Table S8 in Supplementary Material), suggesting a direct role of plasma cells. However, if the analysis was adjusted for intratumoral CD20^+^ B cell density, some of the genes retained significant prognostic impact (Table S9 in Supplementary Material), such as *IGHM* and *IGHG1*.

**Table 5:** Multivariate analysis of expression level of IgG genes and survival outcomes in TNBC patients (after adjustment for for tumor size, grade, age and lymph node status).

	Hazard ratio	95% confidence interval	*p*Value
**Disease-free survival (DFS)**			
*IGKC* (every 1 unit increase of Nanostring count)	0.58	(0.39, 0.88)	0.0103*
*IGHM* (every 1 unit increase of Nanostring count)	0.60	(0.42, 0.86)	0.0055*
*IGHG1* (every 1 unit increase of Nanostring count)	0.66	(0.44, 0.99)	0.0437*
**Overall survival (OS)**			
*IGKC* (every 1 unit increase of Nanostring count)	0.66	(0.41, 1.06)	0.0854
*IGHM* (every 1 unit increase of Nanostring count)	0.65	(0.40, 1.04)	0.0707
*IGHG1* (every 1 unit increase of Nanostring count)	0.64	(0.42, 0.97)	0.0340*

*Statistically significant.

### Intratumoral CD38^+^ Plasma Cell Density and IgG Gene Expression Add Significant Prognostic Power to Classical Clinicopathological Parameters

To test the prognostic power of the B cell/plasma cell-related measures reported here, we evaluated the impact of incorporating their effects into survival outcome analysis with a panel of traditional clinicopathological features (patient age, tumor grade, tumor size, and lymph node status). As shown in Table 6, the additional assessment of intratumoral CD38^+^ plasma cell density with clinicopathological features significantly increased the prognostic value for both DFS (ΔLRχ^2^ = 17.28, *p* = 1.71E−08) and OS (ΔLRχ^2^ = 10.03, *p* = 6.32E−08), compared to clinicopathological features alone. Of the three genes tested (*IGKC, IGHM*, and *IGHG1*), *IGHG1* conferred the highest added prognostic value for both DFS (ΔLRχ^2^ = 21.99, *p* = 1.88E−09) and OS (ΔLRχ^2^ = 16.23, *p* = 3.47E−09). Adding expression level of IgG metagene (Table S7 in Supplementary Material) also increased the prognostic value compared to clinicopathological features alone (DFS: ΔLRχ^2^ = 7.74, *p* = 1.43E−06; OS: ΔLRχ^2^ = 6.12, *p* = 3.89E−07). The best combination was achieved by integrating intratumoral CD38^+^ plasma cell density and *IGHG1* which conferred the best added prognostic value for DFS (ΔLRχ^2^ = 27.38, *p* = 5.22E−10) and OS (ΔLRχ^2^ = 21.29, *p* = 1.03E−08).

**Table 6:** Table showing the change in the log-likelihood of the models with added prognostic terms.

Variables	Disease-free survival		Overall survival	
	ΔLRχ^2^	*p*Value	ΔLRχ^2^	*p*Value
CP + iCD38 PC vs. CP	17.28	1.71E−08*	10.03	6.32E−08*
CP + *IGKC* vs. CP	18.45	9.91E−09*	14.26	8.74E−09*
CP + *IGHM* vs. CP	15.44	4.04E−08*	15.14	5.78E−09*
CP + *IGHG1* vs. CP	21.99	1.88E−09*	16.23	3.47E−09*
CP + IgG genes vs. CP	7.74	1.43E−06*	6.12	3.89E−07*
CP + iCD38 PC + *IGHG1* vs. CP	27.38	5.22E−10*	21.29	1.03E−08*

*Statistical significance of the change was determined by a likelihood ratio test*.

*Statistically significant; CP, clinicopathological parameters (patient age, tumor grade, tumor size, and lymph node status); iCD38 PC, intratumoral CD38^+^ plasma cell density; IgG genes, expression level of a panel of IgG genes (Table S7 in Supplementary Material); LR, likelihood ratio.

## Discussion

While the significance of tumor-infiltrating T cells in breast cancer is well accepted, this is the first study to our knowledge, to provide evidence for a critical role of both B cells and plasma cells in TNBC. Here, we demonstrated that intratumoral CD38^+^ plasma cell density is an independent and incremental prognostic marker, even after adjusting for patient age, tumor grade, tumor size, lymph node status, and the density of tumor-infiltrating CD3^+^ T cells and CD20^+^ B cells. We also report that higher expression level of a panel of IgG genes also predicted better clinical outcome in TNBC patients, supporting a previous publication (44).

Much of the work on B cells and plasma cells in breast cancer has been at the molecular level. Some studies provide evidence that high expression of a B cell/plasma cell metagene is associated with favorable prognosis in breast cancers (43–46); others propose that expression level of *IGKC* alone has equivalent predictive and prognostic value (46). In our hand, expression of *IGHG1*, and not *IGKC*, offered the most significant increases in prognostic value compared to classical clinicopathological parameters alone. Intriguingly, expression level of a subset of the 60-gene B cell/plasma cell metagene is associated with worse prognosis in various cancer types that are reported (43, 67, 68), including the finding that expression of *IGHG1* may be linked with tumor cell proliferation and immune evasion in pancreatic, lung, and breast cancer cell lines (69–72). These data may imply that B cells or plasma cells could assume pro-tumoral roles under certain conditions; however, the factors driving the emergence of this putative pathologic phenotype and the roles played by B cells and plasma cells in these circumstances have yet to be revealed. Of note, studying the *IGHG1* expression may also be valuable if the setting allowed molecular testing such as quantitative polymerase chain reaction (Table 6). We have performed a parallel set of immunohistochemical stains using CD138 for plasma cells in our cohort. CD38 expression in the plasma cells moderately correlated with CD138 in our patient cohort (*R* = 0.39, *p* < 0.0001). Although CD138 has been proposed to also be a plasma cells marker (73), it is widely known that many types of tumor cells can express CD138 including breast cancer (54–57). Since we also observed CD138 strong staining in tumor cells of our TNBC cohort, we did not pursue it further.

The limited cellular level studies on the roles of B cells and plasma cells in breast cancer have generated similar discordant conclusions: Mohammed et al. used IHC staining to show that a high density of CD38^+^ lymphocytes predicted worse prognosis, while the density of CD20^+^ B cells did not significantly affect outcome in primary invasive ductal breast cancer (41). It is possible that in this tumor type, as in esophageal and gastric cancers (74), that immunosuppressive IL10-expressing plasma cells were present and inhibited the anti-tumor T cell response. In this study, we have no evidence of such a pathological role of plasma cells in TNBC. Thus, as suggested by the molecular data, distinct immune mechanisms may be operating in different cancer subtypes and under specific sets of conditions.

The importance of immune parameters in determining prognosis and treatment response across all cancer types has been recognized in several attempts to incorporate their measurement into routine clinical practice (52, 75). However, these attempts have not been successful; moreover both approaches exclude mention of B cells or plasma cells, likely due to the relative lack of data and integrated studies in this area. Our data argue for a re-appraisal of these guidelines and for more widespread investigation of the functional and prognostic roles of these cell types and their gene products.

In summary, our results demonstrate that the density of CD38^+^ plasma cells within TNBC tumors has a significant impact on DFS rate. The prognostic value of plasma cell density is independent of clinicopathological parameters, and of the densities of tumor-infiltrating T cells and B cells. In addition, expression level of the IgG gene, *IGHG1* provided high prognostic value in TNBC for both OS and DFS, representing an easily measurable molecular prognostic marker. The important role of the humoral immune system warrants further studies and may be potentially utilized in routine diagnostic work in addition to its inclusion in cancer immunotherapy.

## Ethics Statement

The Centralized Institutional Review Board of SingHealth provided ethical approval for the use of patient materials in this study (CIRB Ref: 2013/664/F and 2015/2199). In Singapore, a retrospective study is exempted for informed consent.

## Author Contributions

PT and JI conceived and directed the study. PT, JY, and JI supervised the research. JY interpreted the data and performed biostatistical analysis. JL constructed TMAs, performed IHC, prepared samples for NanoString, and collated data. BL performed bioinformatics analysis. HL and WL performed biostatistical analysis. NC and JI performed immunohistochemical scoring. CO constructed TMAs, performed IHC, and collated data. TP and AT contributed to the scientific content of the study. JY and JI drafted the manuscript with the assistance and final approval of all authors.

## Conflict of Interest Statement

The authors declare that the research was conducted in the absence of any commercial or financial relationships that could be construed as a potential conflict of interest.
