# Supplementary material for: High Densities of Tumor-Associated Plasma Cells Predict Improved Prognosis in Triple Negative Breast Cancer
Source: Front Immunol. 2018 May 30;9:1209. doi: 10.3389/fimmu.2018.01209 (PMC5988856; doi:10.3389/fimmu.2018.01209)
Supplement: Supplementary file 1 [file table_1.docx]

Supplementary Material

**High Densities of Tumor-associated Plasma Cells Predict Improved Prognosis in Triple Negative Breast Cancer**

**Joe Yeong^1,2^, Jeffrey Chun Tatt Lim^1^, Bernett Lee^2^, Huihua Li^3^, Noel Chia^4^, Clara Chong Hui Ong^1^, Weng Kit Lye^5^, Thomas Choudary Putti^6^, Aye Aye Thike^1^, Puay Hoon Tan^1^, Jabed Iqbal^1,^***

^1^ Division of Pathology, Singapore General Hospital, Singapore

^2^ Singapore Immunology Network (SIgN), Agency of Science, Technology and Research (A*STAR), Singapore

^3^ Division of Medicine, Singapore General Hospital, Singapore

^4^ Faculty of Medicine, University of New South Wales, Sydney, Australia

^5^ Centre for Quantitative Medicine, Duke-NUS Medical School, Singapore

^6^ Department of Pathology, Yong Loo Lin School of Medicine, National University of Singapore, Singapore

* **Correspondence:**

Jabed Iqbal

Email: [jabed.iqbal@singhealth.com.sg](mailto:jabed.iqbal@singhealth.com.sg), Phone: (65) 6326 5945, Fax: (65) 6227 5945

Address: 20 College Road, Academia, Level 10, Diagnostics Tower, Singapore 169856.

1. **Supplementary Tables**

**Supplementary Table 1**. Comparison of clinicopathological features of the triple negative breast cancers and patients between high and low intra-tumoral and stromal CD38+ plasma cell groups.

|  | **Intratumoral** | | | **Stromal** | | |
| --- | --- | --- | --- | --- | --- | --- |
| **Factor** | **Low CD38^+^ Plasma** | **High CD38^+^ Plasma** | **P value** | **Low CD38^+^ Plasma** | **High CD38^+^ Plasma** | **P value** |
| **Age** |  |  | 0.7029 |  |  | 0.2059 |
| ≤55 | 71 (47.3%) | 47 (44.3%) |  | 90 (44.6%) | 36 (53.7%) |  |
| >55 | 79 (52.7%) | 59 (55.7%) |  | 112 (55.4%) | 31 (46.3%) |  |
| **Ethnicity** |  |  | 0.6520 |  |  | 0.7570 |
| Chinese | 126 (84%) | 84 (79.2%) |  | 163 (80.7%) | 58 (86.6%) |  |
| Indian | 6 (4%) | 7 (6.6%) |  | 11 (5.4%) | 3 (4.5%) |  |
| Malay | 9 (6%) | 6 (5.7%) |  | 13 (6.4%) | 2 (3%) |  |
| Others | 9 (6%) | 9 (8.5%) |  | 15 (7.4%) | 4 (6%) |  |
| **Size** |  |  | 0.4987 |  |  | 0.7624 |
| ≤20 | 52 (35.1%) | 32 (30.5%) |  | 64 (32%) | 23 (34.8%) |  |
| >20 | 96 (64.9%) | 73 (69.5%) |  | 136 (68%) | 43 (65.2%) |  |
| **Associated DCIS** |  |  | 0.7863 |  |  | 0.6202 |
| High | 66 (80.5%) | 46 (85.2%) |  | 79 (80.6%) | 37 (88.1%) |  |
| Intermediate | 15 (18.3%) | 8 (14.8%) |  | 18 (18.4%) | 5 (11.9%) |  |
| Low | 1 (1.2%) | 0 (0%) |  | 1 (1%) | 0 (0%) |  |
| **Tumor histological grade** |  |  | 0.0563 |  |  | 0.2039 |
| 1 | 5 (3.4%) | 1 (0.9%) |  | 4 (2%) | 2 (3%) |  |
| 2 | 25 (16.9%) | 9 (8.5%) |  | 31 (15.4%) | 5 (7.6%) |  |
| 3 | 118 (79.7%) | 96 (90.6%) |  | 166 (82.6%) | 59 (89.4%) |  |
| **Tumor Subtype** |  |  | 0.7037 |  |  | 0.5356 |
| IDC | 130 (86.7%) | 94 (88.7%) |  | 173 (85.6%) | 60 (89.6%) |  |
| Other subtypes | 20 (13.3%) | 12 (11.3%) |  | 29 (14.4%) | 7 (10.4%) |  |
| **Lymphovascular Invasion** |  |  | 0.7928 |  |  | 0.1400 |
| Absent | 95 (63.3%) | 69 (65.1%) |  | 127 (62.9%) | 49 (73.1%) |  |
| Present | 55 (36.7%) | 37 (34.9%) |  | 75 (37.1%) | 18 (26.9%) |  |
| **Lymph Node Stage** |  |  | 0.1600 |  |  | 0.6734 |
| 0 | 75 (63%) | 37 (48.1%) |  | 85 (56.3%) | 36 (64.3%) |  |
| 1 | 25 (21%) | 19 (24.7%) |  | 34 (22.5%) | 12 (21.4%) |  |
| 2 | 12 (10.1%) | 14 (18.2%) |  | 20 (13.2%) | 6 (10.7%) |  |
| 3 | 7 (5.9%) | 7 (9.1%) |  | 12 (7.9%) | 2 (3.6%) |  |

**Supplementary Table 2.** Antibodies used for immunohistochemical (IHC) labeling of TNBC sections.

| **Antibody** | **Clone** | **Dilution** | **Source** | **Labeling Pattern** |
| --- | --- | --- | --- | --- |
| ER | SP1 | 1:50 | Thermo Scientific Lab Vision RM 9101-S | Nuclear |
| PR | SP2 | 1:200 | Thermo Scientific Lab VisionRM9102-S | Nuclear |
| HER2 | SP3 | 1:200 | Thermo Scientific Lab VisionRM9103-S | Membranous |
| CK14 | LL002 | 1:20 | Leica Novocastra NCL-L-LL002 | Cytoplasmic |
| EGFR | E30 | 1:50 | Dako M7239 | Membranous |
| Cytokeratin High Molecular Weight | 34βE12 | 1:200 | Dako M0630 | Cytoplasmic |
| CD38 | SPC32 | 1:50 | NCL-CD38-290 | Membranous |
| CD20 | L26 | 1:200 | Dako M0755 | Membranous and/or cytoplasmic |

**Supplementary Table 3.** Correlation between intratumoural CD38^+^ plasma cells, intratumoural CD20^+^ B cells, RNA level of *IGKC*, *IGHM* and *IGHG1.*

|  | Intratumoural CD20^+^ B cells | *IGKC* gene | *IGHM* gene | *IGHG1* gene |
| --- | --- | --- | --- | --- |
| Intratumoural CD38^+^ plasma cells | p<0.0001*  R=0.558 | p<0.0001*  R=0.647 | p<0.0001*  R=0.580 | p<0.0001*  R=0.655 |

*Statistically significant

**Supplementary Table 4.** Multivariate analysis of B cell/plasma cell density phenotypes and survival outcomes in TNBC patients. (Adjusted for tumor size, histological grade, age and lymph node status)

| **Disease-free survival (DFS)** | | | |
| --- | --- | --- | --- |
| **Stromal CD38^+^ plasma cells TNBCs**  High Vs. Low | **Hazard Ratio** | **95% Confidence Interval** | **P value** |
|  | 0.92 | (0.53, 1.59) | 0.763 |
| **Stromal CD38^+^ plasma cells TNBCs**  (every 1 percent) | 1.00 | (0.98, 1.02) | 0.705 |
| **Stromal CD20^+^ B cells TNBCs**  High Vs. Low | 0.50 | (0.25, 1.00) | 0.049* |
| **Stromal CD20^+^ B cells TNBCs**  (every 1 percent) | 0.98 | (0.96, 1.00) | 0.022* |
| **Overall survival (OS)** | | | |
| **Stromal CD38^+^ plasma cells TNBCs**  High Vs. Low | **Hazard Ratio** | **95% Confidence Interval** | **P value** |
|  | 0.97 | (0.51, 1.86) | 0.938 |
| **Stromal CD38^+^ plasma cells TNBCs**  (every 1 percent) | 1.01 | (0.95, 1.03) | 0.421 |
| **Stromal CD20^+^ B cells TNBCs**  High Vs. Low | 0.34 | (0.15, 0.80) | 0.013* |
| **Stromal CD20^+^ B cells TNBCs**  (every 1 percent) | 0.98 | (0.96, 1.01) | 0.064 |

*Statistically significant

**Supplementary Table 5.** Multivariate analysis of combinatorial stromal B cell/plasma cell density phenotypes with survival outcomes in TNBC patients.

| **Disease-free survival (DFS)** | | | | |
| --- | --- | --- | --- | --- |
| Low stromal CD20^+^ B cell and low stromal CD38^+^ plasma cell TNBCs | **N number**  63 | **Hazard Ratio** | **95% Confidence Interval** | **P value** |
| High stromal CD20^+^ B cell and low CD38^+^ plasma cell TNBCs | 51 | 0.37 | (0.17, 0.80) | 0.012* |
| Low stromal CD20^+^ B cell and high CD38^+^ plasma cell TNBCs | 3 | - | - | 0.997 |
| High stromal CD20^+^ B cell and high CD38^+^ plasma cell TNBCs | 6 | 1.05 | (0.31, 3.57) | 0.943 |
| **Overall survival (OS)** | | | | |
| Low stromal CD20^+^ B cell and low stromal CD38^+^ plasma cell TNBCs | **N number**  63 | **Hazard Ratio** | **95% Confidence Interval** | **P value** |
| High stromal CD20^+^ B cell and low CD38^+^ plasma cell TNBCs | 51 | 0.28 | (0.11, 0.73) | 0.009* |
| Low stromal CD20^+^ B cell and high CD38^+^ plasma cell TNBCs | 3 | - | - | 0.998 |
| High stromal CD20^+^ B cell and high CD38^+^ plasma cell TNBCs | 6 | 0.51 | (0.07, 3.93) | 0.518 |

*Statistically significant

**Supplementary Table 6.** Multivariate analysis of intratumoral CD38^+^ plasma cell density and survival outcomes in TNBC, adjusted for tumor size, histological grade, age and lymph node status and intratumoral CD20^+^ B cell density.

| **Disease-free survival (DFS)** | | | |
| --- | --- | --- | --- |
| **Intratumoral CD38^+^ plasma cell TNBCs**  High Vs. Low | **Hazard Ratio** | **95% Confidence Interval** | **P value** |
|  | 0.25 | (0.10, 0.62) | 0.003* |
| **Intratumoral CD38^+^ plasma cell TNBCs**  (every 1 percent) | 0.94 | (0.90, 0.99) | 0.016* |
| **Overall survival (OS)** | | | |
| **Intratumoral CD38^+^ plasma cell TNBCs**  High Vs. Low | **Hazard Ratio** | **95% Confidence Interval** | **P value** |
|  | 0.23 | (0.08, 0.66) | 0.006* |
| **Intratumoral CD38^+^ plasma cell TNBCs**  (every 1 percent) | 0.95 | (0.91, 1.01) | 0.067 |

*Statistically significant

**Supplementary Table 7.** Log-rank analysis of the association between expression level of IgG genes and survival outcome in TNBC. Median expression levels were used as the threshold for positivity.

| **Official Gene Symbol** | **Full Gene Name** | **P value (OS)** | **P value (DFS)** |
| --- | --- | --- | --- |
| *GUSBP11* | glucuronidase, beta pseudogene 11 | 1.914E−02 | N.S. |
| *IGHA1* | immunoglobulin heavy constant alpha 1 | N.S. | N.S. |
| *IGHD* | immunoglobulin heavy constant delta | N.S. | N.S. |
| *IGHG1* | immunoglobulin heavy constant gamma 1 (G1m marker) | 2.743E−03 | 9.827E−04 |
| *IGHG3* | immunoglobulin heavy constant gamma 3 (G3m marker) | N.S. | 1.361E−02 |
| *IGHM* | immunoglobulin heavy constant mu | 2.877E−02 | 2.472E−03 |
| *IGHV4OR15-8* | immunoglobulin heavy variable 4/OR15-8 (non-functional) | N.S. | N.S. |
| *IGKC* | immunoglobulin kappa constant | 1.662E−04 | 6.355E−05 |
| *IGKV1-37* | immunoglobulin kappa variable 1-37 (non-functional) | N.S. | N.S. |
| *IGKV1D-13* | immunoglobulin kappa variable 1D-13 | 3.294E−03 | 1.475E−04 |
| *IGKV1OR2-108* | immunoglobulin kappa variable 1/OR2-108 (non-functional) | N.S. | 1.012E−02 |
| *IGKV1OR2-118* | immunoglobulin kappa variable 1/OR2-118 (pseudogene) | 9.658E−03 | 1.422E−03 |
| *IGLC2* | immunoglobulin lambda constant 2 (Kern-Oz- marker) | 9.391E−03 | 3.258E−03 |
| *IGLJ3* | immunoglobulin lambda joining 3 | 8.751E−03 | 1.449E−02 |
| *IGLL1* | immunoglobulin lambda-like polypeptide 1 | N.S. | N.S. |
| *IL8* | chemokine (C-X-C motif) ligand 8 | N.S. | 2.020E−02 |
| *POU2AF1* | POU class 2 associating factor 1 | 1.913E−04 | 1.054E−05 |

N.S.: Not significant

**Supplementary Table 8.** Multivariate analysis of expression level of IgG genes and survival outcome in TNBC (adjusted for tumor size, histological grade, age and lymph node status and density of intratumoral CD38^+^ plasma cells).

| **Disease-free survival (DFS)** | | | |
| --- | --- | --- | --- |
| ***IGKC***  (Every 1 unit increase of Nanostring count) | **Hazard Ratio** | **95% Confidence Interval** | **P value** |
|  | 0.77 | (0.47, 1.27) | 0.311 |
| ***IGHM***  (Every 1 unit increase of Nanostring count) | 1.07 | (0.65, 1.76) | 0.790 |
| ***IGHG1***  (Every 1 unit increase of Nanostring count) | 0.78 | (0.51, 1.20) | 0.265 |
| **Overall survival (OS)** | | | |
| ***IGKC***  (Every 1 unit increase of Nanostring count) | **Hazard Ratio** | **95% Confidence Interval** | **P value** |
|  | 0.73 | (0.41, 1.27) | 0.260 |
| ***IGHM***  (Every 1 unit increase of Nanostring count) | 0.76 | (0.43, 1.36) | 0.359 |
| ***IGHG1***  (Every 1 unit increase of Nanostring count) | 0.68 | (0.42, 1.12) | 0.132 |

*Statistically significant

**Supplementary Table 9.** Multivariate analysis of expression level of IgG genes and survival outcome in TNBC (adjusted for tumor size, histological grade, age and lymph node status and density of intratumoral CD20^+^ B cells).

| **Disease-free survival (DFS)** | | | |
| --- | --- | --- | --- |
| ***IGKC***  (Every 1 unit increase of Nanostring count) | **Hazard Ratio** | **95% Confidence Interval** | **P value** |
|  | 0.57 | (0.29, 1.11) | 0.0978 |
| ***IGHM***  (Every 1 unit increase of Nanostring count) | 0.48 | (0.23, 0.99) | 0.0484* |
| ***IGHG1***  (Every 1 unit increase of Nanostring count) | 0.46 | (0.24, 0.87) | 0.0170* |
| **Overall survival (OS)** | | | |
| ***IGKC***  (Every 1 unit increase of Nanostring count) | **Hazard Ratio** | **95% Confidence Interval** | **P value** |
|  | 0.60 | (0.30, 1.44) | 0.298 |
| ***IGHM***  (Every 1 unit increase of Nanostring count) | 0.50 | (0.21, 1.21) | 0.125 |
| ***IGHG1***  (Every 1 unit increase of Nanostring count) | 0.55 | (0.26, 1.15) | 0.113 |

*Statistically significant
